# Spectral divergence prioritizes key classes, genes, and pathways shared between substance use disorders and cardiovascular disease

**DOI:** 10.3389/fnins.2025.1572243

**Published:** 2025-07-22

**Authors:** Everest Castaneda, Elissa Chesler, Erich Baker

**Affiliations:** ^1^Department of Biology, Baylor University, Waco, TX, United States; ^2^School of Engineering and Computer Science, Baylor University, Waco, TX, United States; ^3^The Jackson Laboratory, Bar Harbor, ME, United States; ^4^Department of Mathematics and Computer Science, Belmont University, Nashville, TN, United States

**Keywords:** disease-associated prioritization, substance use disorder, cardiovascular disease, graph spectrum, functional fingerprint

## Abstract

**Introduction:**

Substance use disorders (SUDs) are heterogeneous diseases with overlapping biological mechanisms and often present with co-occurring disease, such as cardiovascular disease (CVD). Gene networks associated with SUDs also implicate additional biological pathways and may be used to stratify disease subtypes. Node and edge arrangements within gene networks impact comparisons between classes of disease, and connectivity metrics, such as those focused on degrees, betweenness, and centrality, do not yield sufficient discernment of disease network classification. Comparatively, the graph spectrum's use of comprehensive information facilitates hypothesis testing and inter-disease clustering by using a larger range of graph characteristics. By adding a connectivity-based method, network rankings of similarity and relationships are explored between classes of SUDs and CVD.

**Methods:**

Graph spectral clustering's utility is evaluated relative to commonly used network algorithms for discernment between two distinct co-occurring disorders and capacity to rank pathways based on their distinctiveness. A collection of graphs' structures and connectivity to functionally identify the relationship between CVD and each of four classes of SUDs, namely alcohol use disorder (AUD), cocaine use disorder (CUD), nicotine use disorder (NUD), and opioid use disorder (OUD) is evaluated. Moreover, a Kullback-Leibler (KL) divergence is implemented to identify maximally distinctive genes (*D*^*g*^). The emphasis of genes with high *D*^*g*^ enables a Jaccard similarity ranking of pathway distinctiveness, creating a functional “network fingerprint”.

**Results:**

Spectral graph outperforms other connectivity-based approaches and reveals interesting observations about the relationship among SUDs. Between CUD and CVD, the gamma-aminobutyric acidergic and arginine metabolism pathways are distinctive. The neurodegenerative prion disease and tyrosine metabolism are emphasized between OUD and CVD. The graph spectrum between AUD and NUD to CVD is not significantly divergent.

**Conclusion:**

Graph spectral clustering with KL divergence illustrates differences among SUDs with respect to their relationship to CVD, suggesting that despite a high-level co-occurring diagnosis or comorbidity, the nature of the relationship between SUD and CVD varies depending on the substance involved. The graph clustering method simultaneously provides insight into the specific biological pathways underlying these distinctions and may reveal future basic and clinical research avenues into addressing the cardiovascular sequelae of SUD.

## 1 Introduction

Complex diseases are caused by a variety of factors and include a range of psychological and physical disorders such as diabetes (Prasad and Groop, 2015), schizophrenia (Sullivan et al., 2003), substance use disorders (Hatoum et al., 2023) (SUDs), cardiovascular disease (CVD) (Musunuru and Kathiresan, 2019), and others (Thaker, 2017; Andrews et al., 2023). These are often co-occurring conditions (Hossain et al., 2020; Solovieff et al., 2013), and due to the many-to-many relationships among genes and disorders (Goh et al., 2007), identifying the specific biological basis of these relationships presents challenges (Wormington et al., 2024). Doing so would enable a refined classification of the particular subtypes of disease that exist and also provide a greater understanding of the nature and mechanism of comorbid disease (Chen et al., 2025; Sánchez-Valle and Valencia, 2023). For example, in various SUDs, there are associations to CVD, but for each drug, the nature of this relationship may differ (Havakuk et al., 2017; Toska and Mayrovitz, 2023; Pando-Naude et al., 2021). There are several approaches to comparing genetic studies to elucidate the nature and extent of these relationships among complex diseases (Gerring et al., 2024). For example, shared genetic liability between SUD and CVD has been found using polygenic risk scores and linkage disequilibrium, but even with shared multimorbid association (Zhou et al., 2017), the shared functional mechanisms are poorly understood (Morgan et al., 2022).

CVD is a leading cause of death and a common multimorbid and comorbid condition, with high prevalence in people with SUD (Chelikam et al., 2022). While the impacts of SUD and CVD concentrate in different tissues, they share similar genetic associations (Hatoum et al., 2023; Sanchez-Roige et al., 2022). Furthermore, the tremendous number of genetic variants impacting the function of the nervous system and heart (Jonker et al., 2023) presents challenges in prioritization of disease-associated genes (Zhukovsky et al., 2024; Guo et al., 2021). A functional enrichment provides foundational interpretation of variant effects at the level of cellular and metabolic processing underlying disease genes (Reimand et al., 2019). Furthermore, the shared risk factors between psychiatric disorders is such that a focus on specific disorders, independent of the context of comorbid and multimorbid conditions, is insufficient for classification (Chen et al., 2025). To compare disorders on a functional level, pathways have been assessed by the intersection of genes, such as network merging for “network fingerprinting”, which has shown that the arrangement of nodes and edges impacts comparisons on similarity scores (Cui et al., 2015). Moreover, the availability of data on a large number of SUDs (Bough and Pollock, 2018; Hatoum et al., 2023; Uffelmann et al., 2021) enables an assessment of the influence between SUD specific functional pathways and a common multimorbid condition, CVD (Minhas et al., 2024). Comparisons between SUDs and cardiometabolic disease provides insight into shared genes, which are highly translatable to therapeutic potential (Sanchez-Roige et al., 2022; Peng et al., 2021).

Investigating and integrating genomic studies of disease can improve disease diagnosis and characterization (Wirka et al., 2018). From genome-wide associations (GWAS) (Uffelmann et al., 2021) to curated database mining (Piñero et al., 2020), discrete experimental investigations of disorders often converge to a functional classification (Reimand et al., 2019). Enrichment software then gauges biological pathways or functional terms that have, more than by random chance, a significant representation (Kuleshov et al., 2016; Wang et al., 2017; Raudvere et al., 2019; Reimand et al., 2019). A functional characterization may focus on a set of a gene's medicinal, cellular, or biological significance (Wieder et al., 2021; Baltoumas et al., 2021). Several databases are used as a proxy for functional analyses, which include the following: the Kyoto Encyclopedia of Genes and Genomes (KEGG) (Kanehisa and Goto, 2000; Kanehisa, 2019; Kanehisa et al., 2023), WikiPath (Agrawal et al., 2023), Gene Ontology (GO) (Ashburner et al., 2000; Consortium et al., 2023), and Reactome (Milacic et al., 2023), among others (Geistlinger et al., 2021; Zhao and Rhee, 2023).

Spectral graph analysis presents a promising approach to simultaneously compare disease based on the various genomic data sources and to identify the biomolecular pathways that can be used to classify them. Graph spectrum has been used for hypothesis testing (Takahashi et al., 2012; Fujita et al., 2017), differentiating diseases and tissues (Santos et al., 2015; Jardim et al., 2019), identifying functional pathways (Fujita et al., 2017), and clustering (Sato et al., 2013) in neurological disorders. The spectrum of a graph contains information on several important dynamics such as number of walks, diameter, and cliques (Takahashi et al., 2012); therefore, the spectrum is more informative to characterizing complex networks than modern metrics (Fujita et al., 2017). Contemporary approaches have classified genes by importance through nodal connectivity evaluations and pinpointed dysfunction or distinguished biological conditions (Barabási and Oltvai, 2004; Gu et al., 2012; Rahmatallah et al., 2013; Santos et al., 2015).

Here, we present a functional analysis of five complex diseases describing the genes underlying 4 SUDs and CVD, a commonly co-occuring condition (Minhas et al., 2024). Moreover, we attempt to elucidate key differences in classes of SUDs and their associations to CVD, resulting in the prioritization of key disease-genes and pathways through graph spectral clustering, which relies on graph structure is and not limited to genomic intersections. We conduct a graph spectral analysis of SUD and CVD related genomic studies with functional KEGG pathways using the statGraph (Castro Guzman et al., 2024) package and posit a comparative insight against other connectivity indices (Fujita et al., 2017) using high fidelity pathways created by KNeXT (Castaneda and Baker, 2024). By leveraging the topological information, the arrangement of nodes and edges, offered by KEGG graphs, we demonstrate that the spectral distribution aids in defining key divergences, or its absence, between four of the following SUDs: AUD, CUD, OUD, and NUD with its comorbidity, CVD. Moreover, we elucidated the biological relevance of principle divergent-driving elements and outline functional class differences between CUD and OUD.

## 2 Materials and methods

### 2.1 Datasets

**Figure 1:**
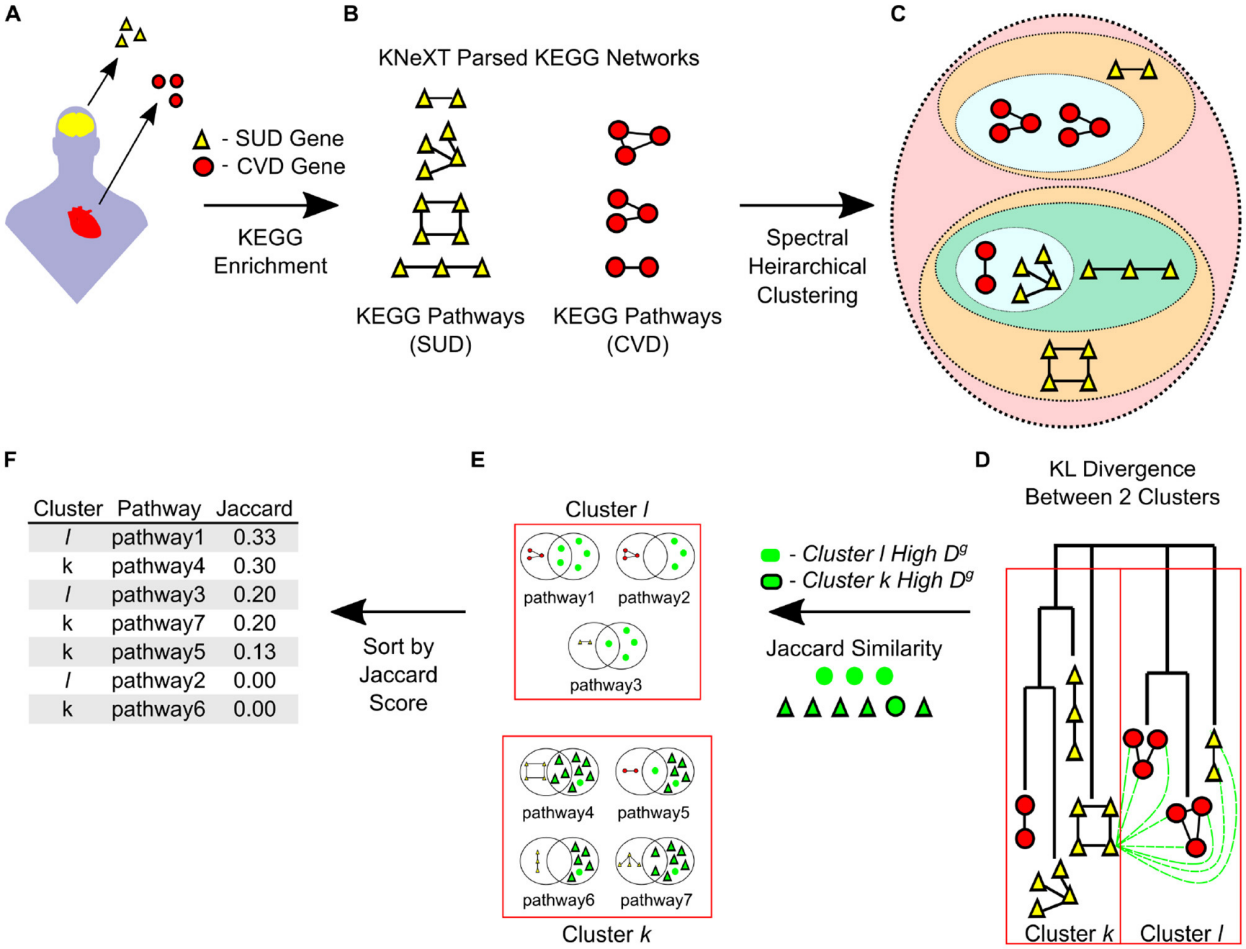
The framework of prioritizing pathways and genes using spectral clustering and a KL divergence. **(A)** Disorder-associated genes sets derived from humans, acquired from DisGeNET and published sources, are prioritized from experimental analyses or from database mining and are subsequently enriched for KEGG pathways. **(B)** Enriched pathways' KGML files are then parsed in KNeXT as functional gene-gene networks. **(C)** KNeXT-generated gene networks are hierarchically clustered through spectral clustering. **(D)** Post clustering, individual genes are assessed through KL divergence against an opposing cluster, dotted green lines. **(E)** Genes in cluster *k* are compared to genes in cluster *l* and then the comparison is reversed where genes in cluster *l* are compared to genes in cluster *k*. All genes with high *D*^*g*^ are compared to all pathways within its origin cluster. **(F)** The results of this framework are Jaccard scores for all pathways in each cluster. KEGG pathways with a high Jaccard score have an abundance of top *D*^*g*^ genes, which in turn, is driving distinction between clusters.

*Homo sapiens* gene sets were gathered from publicly available repositories and published sources (Figure 1A). Data were collected from DisGeNET (Piñero et al., 2020) using the Harmonizome web service (Rouillard et al., 2016). DisGeNET has been widely used as a benchmark database (Barua et al., 2022; Gentili et al., 2022) due to its comprehensive and curated information on gene-disease associations (Piñero et al., 2020). For each SUD, we used affiliated search terms, noting that the current terminology for CUD has evolved but is not always used in existing data repositories (Deak and Johnson, 2021). These terms include the following: “alcohol use disorder” for AUD, “cocaine abuse” [*sic*] for CUD, “nicotine dependence” for NUD, and “cardiovascular pathology” for CVD. We combined “heroin dependence”, “opioid use disorder”, and “morphine dependence” for our OUD gene set, see Supplementary Table S1 for full list. Genes were then uploaded to g:Profiler, ignoring ambiguous gene queries, which was set to a g:SCS (Set Count and Sizes) threshold of 0.05. Published KEGG CVD pathways were gathered from (Barua et al., 2022), which used Gene Expression Omnibus microarray datasets for assessing CVD and all its risk factors. Neurological KEGG pathways were acquired using the following Biological Relation Inference and Classification Engine (BRITE) terms: *Nervous system, Substance dependence*, and *Neurodegenerative diseases*. Additionally, we included the pathway *Neuroactive ligand-receptor interaction*. All neurological pathways derived from BRITE terms were used for systematically characterizing synapses across regions of the brain (Bar-Shira et al., 2015).

### 2.2 KEGG networks

Here, we focus on KEGG's database which hosts a series of biological systems maps that offer specific molecular pathways based on highly curated and experimentally verified gene-gene, gene-compound, and gene-pathway interactions (Kanehisa and Goto, 2000; Kanehisa, 2019; Kanehisa et al., 2023). KEGG is an important resource because all molecular associations are stored for secondary parsing and analyzing in a standard language: KEGG Markup Language (KGML). KGML files can be readily parsed and used with robust software packages including the following: KEGG NetworkX Topological (KNeXT) Parser (Castaneda and Baker, 2024), graphite (Sales et al., 2012), KEGGParser (Arakelyan and Nersisyan, 2013), among others (Nersisyan et al., 2014; Chanumolu et al., 2021). KNeXT, in particular, focuses on the spatiotemporal dynamics reflected in a KGML file to create high fidelity pathways (Castaneda and Baker, 2024). All KEGG pathways were parsed using the KNeXT parser, see Figure 1B. KNeXT creates high fidelity genes-only pathways (Castaneda and Baker, 2024). For simplicity, all pathways used NCBI gene identifiers, contained no compounds, and are unweighted and undirected. For full list of all KEGG pathways, see Supplementary Table S2.

### 2.3 Spectral discrimination

In this study, we used the statGraph (Castro Guzman et al., 2024) package in R version 4.3.1 (R Core Team, 2023). statGraph features several tests for conducting spectral analyses of graph lists. From this package, we used the Analysis Of Graph Variability (*anogva*), Takahashi Test (*takahashi.test*), and heirarchical clustering (*hclust*). *anogva* performs a statistical test on a set of two or more graphs to determine if they are generated by the same random process (Fujita et al., 2017). *takahashi.test* conducts a statistical test to determine if two sets of graphs are generated by the same random process (Takahashi et al., 2012; Fujita et al., 2017). All tests used a seed set at one. *hclust* conducts a hierarchical clustering of a list of graphs based on their spectral distribution (Figures 1C, D). We used default parameters, which include *complete* agglomerative clustering method with Silverman bandwidth and exact spectral density.

### 2.4 Algorithmic comparisons

For baseline comparisons, we used common indices, which included the following: degree, average betweenness centrality, and closeness centrality (Fujita et al., 2017; Zito et al., 2021). Implementation of these metrics was through NetworkX (Hagberg et al., 2008). We used the Jensen-Shannon (JS) distance in the SciPy version 1.5.0 package (Virtanen et al., 2020) to create distance matrices for input into the *AgglomerativeClustering* function in the Scikit-learn package (Pedregosa et al., 2011). The same parameters to the *hclust* package were used with *complete* linkage.

### 2.5 Statistical comparisons

Statistical comparisons for graph performance was measured using the Adjusted Rand Index (ARI) (Warrens and van der Hoef, 2022). For R analyses, we used the *fossil* package version 0.4.0 (Vavrek, 2011), and for Python analyses, we used the *Scikit-learn* package (Pedregosa et al., 2011). Both metrics measure the accuracy of clustering with ARI being adjusted for randomness. An ARI ≤ 0 is equivalent to random assignments (Yeung et al., 2003). ARI has been used for comparisons of clustering performance in previous works (Wu and Wu, 2020; Zelig et al., 2023).

### 2.6 Gene and pathway prioritization

To prioritize genes and pathways in biological clusters, we modified a method developed by Dey et al. (2017), which uses KL divergence to compare the distinctiveness of a gene, *g*, with respect to any cluster *l* see Equation 1. Here, we used the entropy function in the *Scikit-learn* package (Pedregosa et al., 2011).


(1) $$\begin{array}{l} KL^{g}\left[k,l\right]=\underset{x\in X}{\sum }p^{S_{k}}\left(x\right)\times log\frac{p^{S_{k}}\left(x\right)}{p^{S_{l}}\left(x\right)} \end{array}$$

Let *S* = {*S*_1_, *S*_2_, *S*_3_, ...*S*_*n*_} be a collection of KEGG pathways in cluster *k* with vertex set, *X*. *p* is the degree distribution for any *X* in any pathway in *S* compared to any *X* in any pathway in cluster *l*. Thereby, for each cluster *k*, we measure the distinctiveness of each gene as the minimum divergence (Equation 2).


(2) $$\begin{array}{l} D^{g}\left[k\right]=\underset{l\neq k}{\min}KL^{g}\left[k,l\right] \end{array}$$

Thereby, genes with a maximum distinctiveness (*D*^*g*^) are the genes with the largest role in distinguishing cluster *k* from cluster *l*. After identifying the genes with the highest *D*^*g*^ we use a Jaccard index to determine the set similarity (Equation 3). *D*_*k*_ is the set of genes with the highest *D*^*g*^ in cluster *k* and *S*_*k*_ is some pathway in cluster *k*, see Figures 1D–F.


(3) $$\begin{array}{l} J\left(D_{k},S_{k}\right)=\frac{\left|D_{k}\cap S_{k}\right|}{\left|D_{k}\cup S_{k}\right|} \end{array}$$

## 3 Results

### 3.1 Comparisons of KEGG enriched pathway from DisGeNET derived gene sets

**Figure 2:**
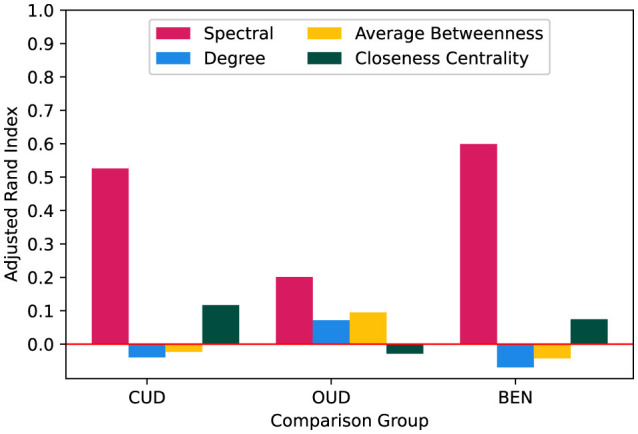
Comparison of spectral clustering to commonly used algorithms. DisGeNET-derived pathway groups include cocaine use disorder (CUD) and opioid use disorder (OUD) compared to cardiovascular disease (CVD). Benchmarked groups (BEN) are groups derived from BRITE terms for nervous system classes and surveyed CVD pathways. Spectral clustering outperformed all other algorithms.

Our first analysis was to determine the significance of the divergence between the four SUDs and CVD KEGG pathway lists. Here, divergence refers to the disparate random processes underlying a collection of graphs as defined by Takahashi et al. (2012); Fujita et al. (2017). We compared each KEGG pathway list derived from highly supported DisGeNET (Piñero et al., 2020) gene sets. First, we used *anogva* to test the spectrum of all data. *anogva* controls for Type I errors and is robust for unbalanced data (Fujita et al., 2017). The results showed a significant JS divergence between all five sets of graphs, see Table 1. Moreover, Takahashi's Test revealed a significant JS divergence between CUD and OUD against CVD but not for AUD and NUD, see Table 1, which parallels past epidemiological studies signifying the strong relationship between AUD, NUD, and CVD (Yeates et al., 2015). Post Takahashi's Test, we then conducted a hierarchical agglomerative clustering on the significant SUDs. Clustering quality was determined by the ARI against commonly used algorithms (Fujita et al., 2017). CUD had the highest ARI and outperformed all other connectivity metrics, and OUD generated the highest ARI compared to baseline measures (Figure 2).

**Table 1:** Results of test for the JS divergence between groups.

**Source**	**Comparison**	**JS**	***p***
DisGeNET	All^a^	< 0.001	**<** **0.001**
		AUD vs CVD	0.016	0.39
		CUD vs CVD	0.027	**0.005**
		NUD vs CVD	0.030	0.12
		OUD vs CVD	0.021	**0.036**
**Benchmarked**	Brain^b^ vs. CVD^c^	0.022	**0.038**

^a^All refers to AUD vs CUD vs CVD vs NUD vs OUD. ^b^Published in Bar-Shira et al. (2015). ^c^Published in Barua et al. (2022). Benchmarked: KEGG pathways used in published studies. Bolded values emphasize statistical significance.

### 3.2 Comparisons of benchmarked KEGG pathways

From the perspective of KEGG pathway enrichment, there are several factors that may generate inconsistent pathway inclusions (Mubeen et al., 2022). In order to show the inherent functional divergence between neurobiologically-derived gene sets and CVD, we conducted an analysis on what we term “benchmarked” KEGG pathways. Pathways are retrieved from published surveys or extensive analyses which focused on KEGG function and require no enrichment profiling. Additionally, these pathways have been used or compared to benchmark data (Bar-Shira et al., 2015; Barua et al., 2022). Takahashi's test illustrates that KEGG pathways involved in the brain are significantly divergent from KEGG pathways involved in CVD, see Table 1. Furthermore, benchmarked KEGG pathways scored the highest ARI of all other pathways (Figure 2). Hence, evidence suggests that the sub-network of KEGG pathways involved in the brain are structurally different when compared to CVD, which is captured by the graph's spectrum.

### 3.3 Analysis of top driving genes

**Figure 3:**
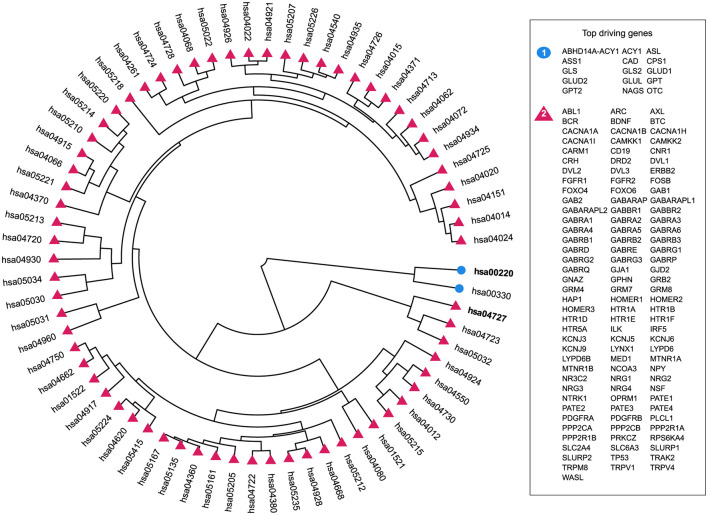
Agglomerative hierarchical clustering for CUD vs. CVD. The top driving genes are genes that have a high *D*^*g*^ and are listed according to their cluster, which is color and shape coordinated. Furthermore, pathways with a high Jaccard index are bolded. Pathway hsa00220 is arginine biosynthesis and hsa04727 is GABAergic synapse.

For OUD and CUD, the two SUDs that significantly differed in divergence, we used the highest *D*^*g*^ (Dey et al., 2017) of each cluster to create gene sets which in turn were used to rank each pathway by their composition using Jaccard similarity. The results of an agglomerative clustering between CUD and CVD illustrate two pathways, hsa00220 (arginine biosynthesis) and hsa00330 (arginine and proline metabolism), are divergent from the rest of the maps, see Figure 3. The top driving genes created gene sets that had the highest similarity with arginine biosynthesis in cluster one and gamma aminobutyric acid (GABAergic) synapse in cluster two (Figure 3).

**Figure 4:**
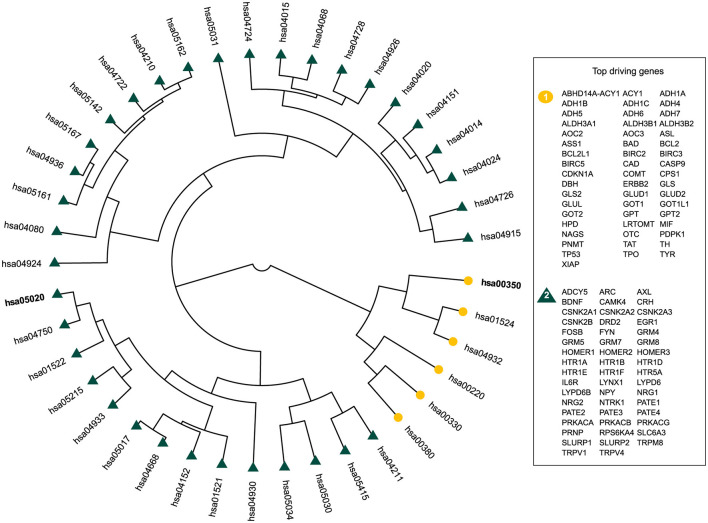
The results of the agglomerative hierarchical clustering for OUD vs. CVD. The top driving genes are genes which have a high *D*^*g*^ and are listed according to their cluster, which is color coordinated. Furthermore, pathways with a high Jaccard index are bolded. Pathway hsa03050 is tyrosine metabolism and pathway hsa05020 is prion disease.

From the top driving genes in OUD vs CVD, the pathways with the highest *D*^*g*^ defined tyrosine metabolism and prion disease (Figure 4). In addition, the top driving genes belonging to the GABAergic synapse in CUD are similarly reflected in the benchmarked gene sets with Glutamatergic synapse, another organismal/nervous system pathway, being highly divergent (Supplementary Figures S1, S2). Given that NF-kappa B signaling pathway was a top driving pathway whose class is not similar to any SUD, this implies an inherit distinctiveness between classes of SUD and the brain in their comparison to CVD (Supplementary Figures S1, S2).

### 3.4 Functional fingerprint

**Figure 5:**
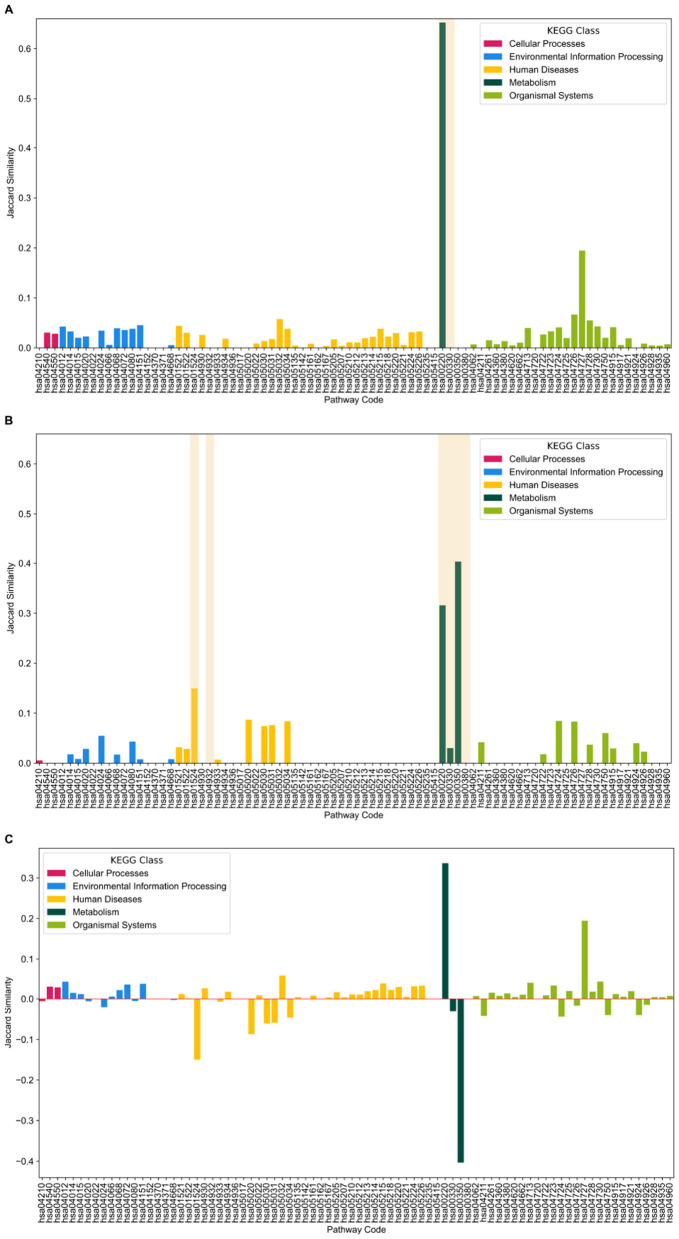
All similarity results sorted by KEGG class. Pathways that cluster separately are highlighted in yellow. **(A)** Similarity results for CUD. As shown, two metabolism pathways diverged compared to the rest of the results. **(B)** Similarity results for OUD. For OUD, metabolism and two human disease pathways drove the cluster separation. **(C)** The differences in magnitude of Jaccard similarity between CUD and OUD. As shown, metabolism plays the largest role in the differences between both CUD and OUD.

We illustrate a “network fingerprint”, as described in Cui et al. (2015) between SUDs and CVD (Figure 5). CUD and OUD, Figures 5A, B, respectively, differ based on the pathways included in each cluster. Furthermore, the division is solely in metabolic pathways in CUD, Figure 5A, while OUD differs in both human diseases and metabolism (Figure 5B). A comprehensive difference was generated between CUD and OUD (Figure 5C). An interesting aspect is the role metabolism plays in both CUD and OUD's largest magnitude of difference, see Figure 5C.

## 4 Discussion

Here, we demonstrated the utility of graph spectral clustering for differentiating between the bases of comorbidity of disease. The technique outlined reveals the effectiveness of assessing the distinctions between classes of SUDs and a commonly co-occurring disease, CVD. We have shown this method to outperform other commonly used algorithms in classifying KEGG pathways derived from SUDs and CVD gene sets. Furthermore, we leveraged spectral clustering to rank pathways according to their distinctiveness between the two conditions, revealing a “network fingerprint” comparison, similarity described in Cui et al. (2015). Our analysis pipeline finds differences among disorders and identifies key pathways, which may have therapeutic or diagnostic consequences. The facilitation of a “network fingerprint” diagram aids in hypothesis building and identifying key functional pathways. From these clusters, we can characterize the associations between two diseases, which are unmatched by KEGG pathway lists alone and other topology-based methods.

KEGG is an important tool for disease study from a functional perspective (Kanehisa and Goto, 2000; Kanehisa, 2019; Kanehisa et al., 2023). Surveys of KEGG pathways and disease interactions infer novel association of overlapping risk factors (Barua et al., 2022) and common disorders, (Li et al., 2008) given sets of prioritized genes (Cirincione et al., 2018). Moreover, other analyses rank and prioritize pathways by significance levels (Chu et al., 2024). While these approaches are useful to generate new insights in highly related diseases that have strong pathway sharing within tissues, they do not provide adequate discernment for two convergent disorders involving different tissues, which share common and distinct biomarkers (Moon et al., 2025; Riley et al., 2022; Gu et al., 2021; Daneshafrooz et al., 2022). Here, we have emphasized pathways through graph connectivity, which does not rely merely on pathway member composition. Hence, we propose future usage for comparisons between disorders that exist in different tissues and experience limited functional overlap, such as certain comparisons of fibrotic disease (Gu et al., 2021) and frontotemporal dementia (FTP) and amyotrophic lateral sclerosis (ALS) (Daneshafrooz et al., 2022). While epidemiological studies demonstrate a strong comorbidity between SUDs, defined here as AUD, NUD, CUD, and OUD, and CVD (Gan et al., 2021; Chelikam et al., 2022), our approach distinguishes the more granular separation of SUDs and CVD based on their KEGG pathway representations. We provide evidence to show that CUD and OUD are significantly divergent to CVD while AUD and NUD are not. This divergence may be explained by differences in how these substances interact with the cardiovascular system (Havakuk et al., 2017; Toska and Mayrovitz, 2023; Jalali et al., 2021) and areas of the brain (Pando-Naude et al., 2021). Additionally, we highlight the specific metabolic and neurological pathways and genes driving these distinctions. The profile of these clusters would be useful in disease state transition surveilling (Guo et al., 2021) and model organism testing. For example, knockouts of genes involved in these pathways may show insights for vulnerabilities to CVD for a given SUD (Cacheiro et al., 2023).

While the main focus of this work was the divergence created by each KEGG pathway, network merging is a crucial aspect of heterogeneous graph development where integrating and comparing graphs is essential (Chang et al., 2016; Zitnik et al., 2024). Moreover, existing software analyses have limited scalability on large data sets (Chang et al., 2016; Smedley et al., 2015). The technique examined here may be applicable for automated KEGG enrichment data set preprocessing, trimming, and curation (Orouji et al., 2024).

Biologically, a synergism exists between the representative genes from pathways with several high *D*^*g*^ genes. For example, tyrosine metabolism disruption (Rathor and Ch, 2023) and OUD is known to affect circadian rhythms (Puig et al., 2023). Neurodegeneration-related pathways are linked to OUD-mediated circadian rhythm disruption (Puig et al., 2023). The prioritized pathways might imply novel transitory genes that are implicated in circadian rhythm disruption as several glutaminergic synaptic signaling genes were prioritized alongside the implications of aromatic amino acid metabolism (Humer et al., 2020; Puig et al., 2023). Moreover, “network fingerprinting” (Cui et al., 2015) clustered prion disease with Type 2 diabetes and amyotrophic lateral sclerosis, which implicates neurodegeneration playing a role in complex diseases. In our CUD clusters, arginine has been studied for its role in CVD prevention and treatment (Tousoulis et al., 2007; Bahadoran et al., 2016). GABA plays a role in both CVD (Bu et al., 2021) and CUD (Wydra et al., 2024). GABA has shown promise as pharmacotherapy for addiction (Wydra et al., 2024), and accordingly, arginine has been shown to synaptically interact with GABA in the brain of rats (Shen et al., 1997). The synergism of these pathways and their divergent-driving genes might have implications in co-occurring (Stoychev et al., 2021) CVD and CUD treatment and study (Wilson et al., 2001).

A limitation of this study is the statistical tests in statGraph do not account for multiple group memberships. Hence, diseases with high overlap of KEGG pathways will create difficulties in using the tools outlined in this analysis. An additional limitation of this study is the high redundancy of KEGG pathways (Karp et al., 2021), which creates issues in finding differences in topology, gene-gene connectivity, as suggested by low ARI scores. Moreover, several software exist for KEGG enrichment (Mubeen et al., 2022), and the pathway database itself may be biased to understudied genes, such as non-coding RNA genes (Li et al., 2022). Consequently, the topology and genomic composition of KEGG pathways are not comprehensive (Wilk and Braun, 2017; Gable et al., 2022). While a survey of all enrichment software and KEGG parsers is beyond the scope of this article, we note that use of different combinations of software and thresholds may produce varying results. Moreover, discrete combinations of search terms for disorders in DisGeNET may yield larger or smaller gene sets, which would render disparate amounts and combinations of KEGG pathways. Hence, the use of benchmarked data served solely to indicate an inherent divergence in pathways representing the brain (Bar-Shira et al., 2015) and pathways underlying a comprehensive study of CVD (Barua et al., 2022).

We have demonstrated how underexplored network features (Santos et al., 2015) may be employed to prioritize or differentiate disorders. In previous functional studies, SUDs are coalesced (Li et al., 2008), which overlooks underlying differences. We leveraged the divergence of the collection of KEGG graphs to prioritize genes that are implicated in driving the functional clustering between SUD and CVD. The pathway and genes prioritized are biologically relevant and might have implications for future studies in knockout or other experimental analyses. Additionally, the pathway and gene rankings could justify inclusion or exclusion in large-scale or heterogeneous network analyses of multiple disorder studies (Xiong et al., 2019; Gu et al., 2022). Furthermore, the magnitude of the pathway ranking differences decomposes the complexity of a collection of KEGG graphs, conferring critical visualization and processing where KEGG lists alone cannot provide.

The graph spectrum reveals a distinction among disorders that are co-occurring and can allow visualization of the relationships among multiple disorders simultaneously. Spectral clustering outperformed other commonly used algorithms in classifying clusters of a psychiatric disorder and a common multimorbidity or comorbidity in CVD, and thus its application to other comorbidities observed in SUDs, psychiatric disorders, and other complex disease is promising. Furthermore, the method can characterize the pathways that drive each cluster's distinction to reveal insights about their biological implications, potential diagnostic, and therapeutic targets. In contrast to many pathway overlap approaches that rely on data from disease that involve a limited tissue or cell population, the method introduced here has implications for identifying genes that drive co-morbid conditions in distinct diseases encompassing a diverse range of tissues and embodying systems networks that have little functional pathway overlap.

## Data Availability

The datasets presented in this study can be found in online repositories. The names of the repository/repositories and accession number(s) can be found in the article/supplementary material.
