# Supplementary material for: Spectral divergence prioritizes key classes, genes, and pathways shared between substance use disorders and cardiovascular disease
Source: Front Neurosci. 2025 Jul 22;19:1572243. doi: 10.3389/fnins.2025.1572243 (PMC12321874; doi:10.3389/fnins.2025.1572243)
Supplement: Supplementary file 1 [file Data_Sheet_1.pdf]

Supplementary Material

Table S1: All genes used in enrichment analysis

disorder	gene
aud	TLR4
aud	CYP2E1
aud	GABRG2
aud	MTA2
aud	NPSR1
aud	CD1D
aud	CALM1
aud	XDH
aud	CHI3L1
aud	FGF2
aud	ACACA
aud	EZH2
aud	GLP1R
aud	PCSK9
aud	ACTB
aud	CD38
aud	TNF
aud	HTR2A
aud	SHBG
aud	VWF
aud	MAP6
aud	OPRM1
aud	GDAP1
aud	CHRNA3
aud	SLC6A3
aud	MCM5
aud	SMR3A
aud	PICALM
aud	BCS1L
aud	DRD3
aud	HDAC1
aud	SORCS2
aud	PDYN
aud	QRSL1
aud	EXT1
aud	CCKAR

aud	TTC12
aud	PDE4B
aud	TLR3
aud	CXCL10
aud	AHR
aud	CNR1
aud	NR3C1
aud	ADRA1D
aud	COX2
aud	TWIST1
aud	ARSI
aud	GRIN2B
aud	DRD4
aud	GPR39
aud	CNTN6
aud	CRP
aud	BAG3
aud	APOL2
aud	GLI2
aud	NLRP3
aud	CHRNB3
aud	GHSR
aud	TLR6
aud	TLR2
aud	TBX19
aud	GRM5
aud	NMUR2
aud	GGT1
aud	DMTN
aud	TACR1
aud	HCRT
aud	SNAP91
aud	TNFSF10
aud	CCL2
aud	NLN
aud	ERCC1
aud	PER2
aud	PKD2L1
aud	CNTN5
aud	TAS2R16
aud	HTR3B
aud	SNCA
aud	AVP

aud	P2RX4
aud	COMT
aud	ADH1A
aud	RAC1
aud	WASF1
aud	ALDH2
aud	IL1B
aud	PNOC
aud	ALK
aud	NMU
aud	CAPS
aud	CRH
aud	PIP4K2C
aud	ALDH1A1
aud	PTGS2
aud	FSHB
aud	PDE4A
aud	PRKCI
aud	FLT4
aud	GPT
aud	BDNF
aud	COPE
aud	S100B
aud	MGC57346-CRHR1
aud	BHLHE41
aud	SLC6A4
aud	CALM2
aud	CDS1
aud	NR1D1
aud	FGFR1
aud	ELK3
aud	TSPO
aud	EBPL
aud	GLO1
aud	OPRK1
aud	PPM1G
aud	ADH1C
aud	HDAC9
aud	PIP5K1C
aud	PECAM1
aud	FKBP5
aud	TAS2R13
aud	LTBP3

aud	ACE
aud	HMGB1
aud	NPY
aud	CXCL8
aud	ARNTL2
aud	TF
aud	ARHGAP22
aud	ADRA1A
aud	DBT
aud	SMS
aud	CAST
aud	NTF3
aud	LHB
aud	CRHR1
aud	NOL3
aud	WASF2
aud	GPHN
aud	APOBEC3C
aud	OPRL1
aud	PPARA
aud	GABRA2
aud	HTR3A
aud	FUS
aud	CCR2
aud	ADH4
aud	CDK20
aud	TAS2R38
aud	GABRA1
aud	GAL
aud	LXN
aud	LEP
aud	TAC1
aud	DRD2
aud	CHRNA4
aud	TACR3
aud	TPH2
aud	MTHFR
aud	CALM3
aud	CLMP
aud	TMEM97
aud	CCL11
aud	XIAP
aud	ADH1B

aud	OXTR
aud	HTR7
aud	CXCL12
aud	RAB3D
aud	BTG3
aud	HTR4
aud	ST18
aud	IAPP
aud	IGF1
aud	PIK3R1
aud	CHRNA5
aud	ZNF469
aud	CYP2D6
aud	GABBR1
aud	TPH1
aud	OPN4
aud	THOP1
aud	IFNG
aud	GATA4
aud	CRHBP
aud	ANKK1
aud	CARTPT
aud	UMOD
aud	MNAT1
aud	DRD1
aud	CPSF4
aud	SAT1
aud	CLOCK
aud	LGALS1
aud	AKR1A1
aud	IL6
aud	DDX53
aud	GABRG1
aud	REM1
aud	NPY2R
aud	NRGN
aud	IL10
aud	SLC29A1
aud	BMS1
aud	TCN1
aud	GDNF
aud	CHM
cud	ADRBK2

cud	AGO2
cud	CES2
cud	CCR5
cud	OR3A2
cud	CNR1
cud	RHOA
cud	CHRNA3
cud	ESR2
cud	GCC1
cud	CRHR1
cud	NCS1
cud	LRRC7
cud	BDNF
cud	EGR1
cud	HTR1A
cud	GRM2
cud	PDYN
cud	C1QL2
cud	GAD2
cud	GPHN
cud	MAOA
cud	EXTL3
cud	SMARCA4
cud	ABCB1
cud	CREB1
cud	DRD2
cud	KLHL5
cud	SLC1A2
cud	F2RL3
cud	PIK3CD
cud	CLOCK
cud	KCTD20
cud	TYK2
cud	GRIA2
cud	SRCIN1
cud	BRD4
cud	NPY2R
cud	GAD1
cud	SMAD5
cud	CHRM5
cud	CACNA1D
cud	CHRNB4
cud	TAAR1

cud	STK38
cud	FGF2
cud	RPS6KA5
cud	SMUG1
cud	TLR3
cud	COMT
cud	FOS
cud	GDNF
cud	POMC
cud	NR3C1
cud	CES1
cud	BCHE
cud	BAZ1A
cud	MAP2K1
cud	MGC57346-CRHR1
cud	ATP2C2
cud	CHRNA5
cud	SIGMAR1
cud	TLR4
cud	LEP
cud	OPRL1
cud	TACR3
cud	SLC6A4
cud	NTNG2
cud	GLI2
cud	SLC18A2
cud	CAMK2B
cud	MANEA
cud	ARC
cud	CHRNB3
cud	CALM1
cud	FAM53B
cud	ADRA1A
cud	DRD3
cud	SMAD1
cud	OPRK1
cud	NTNG1
cud	CARTPT
cud	CDK5
cud	SLC6A3
cud	NGF
cud	HTR2A
cud	MAPK1

cud	NFAT5
cud	SMAD3
cud	CALM2
cud	CRHR2
cud	ANKK1
cud	RUNX2
cud	NPS
cud	ADRB2
cud	ILK
cud	TENM3
cud	EHMT2
cud	ATP9B
cud	HTR1B
cud	GABRG2
cud	PRL
cud	GABBR1
cud	OPRD1
cud	NLN
cud	OPN4
cud	FOSB
cud	ARRB2
cud	CD160
cud	PER1
cud	CCKBR
cud	NTRK2
cud	DRD4
cud	PER2
cud	ICE2
cud	PRMT6
cud	DNAH8
cud	GALR1
cud	SYT13
cud	PNOC
cud	HCRT
cud	DBH
cud	SMURF1
cud	GHRL
cud	CCSER1
cud	DRD1
cud	GABRA2
cud	CAMK2A
cud	CALY
cud	HOMER1

cud	MAPK3
cud	PIK3CB
cud	GRM5
cud	SCARB1
cud	JUND
cud	CAMK4
cud	HOMER2
cud	FDFT1
cud	HMGB1
cud	NOL3
cud	OPRM1
cud	CRHBP
cud	PAWR
cud	UMOD
cud	NPY
cud	PIK3CA
cud	TAC1
cud	HCRTR1
cud	KALRN
cud	RGS4
cud	CHRM4
cud	APOL2
cud	GRIN1
cud	SST
cud	COL21A1
cud	TPH2
cud	SPINT2
cud	CRH
cud	HDAC4
cud	HTR2C
cud	PIK3CG
cud	NCOR2
cud	MC4R
cud	GRIA1
cud	GPSM1
cud	WDHD1
cud	SNCA
cud	CHRNA6
cud	HDAC5
cud	NSF
cud	CDNF
cud	MECP2
cud	AVP

cud	GSTP1
nud	APBB1
nud	CCAR1
nud	GM2A
nud	GRIA4
nud	CACNA2D3
nud	GABRA4
nud	AKR1A1
nud	TYRP1
nud	SEMA6A
nud	PTEN
nud	TAC1
nud	DRD3
nud	CAMK4
nud	CCK
nud	CPD
nud	HCRTR2
nud	CHRNA1
nud	CARTPT
nud	HCRT
nud	NR3C1
nud	SLC6A4
nud	CHRM2
nud	TAS2R38
nud	SLC18A2
nud	ASCC1
nud	CHRNA9
nud	ARHGEF7
nud	IKBKB
nud	ARRB1
nud	NRXN1
nud	CD40
nud	OR2AG1
nud	DLG4
nud	CNR1
nud	AKR1B10
nud	CHRNB3
nud	DRD2
nud	CRH
nud	TPH1
nud	GRIN3A
nud	PLA2G1B
nud	PPP1R1B

nud	DRD1
nud	SLC6A3
nud	CRP
nud	CHAT
nud	NPY2R
nud	GABRE
nud	GAL
nud	CYP2E1
nud	NCAM1
nud	CHRND
nud	RAPGEF4
nud	IL1B
nud	RGS2
nud	CHRNA2
nud	HINT1
nud	ARRB2
nud	GSTM1
nud	KANK1
nud	EGLN2
nud	DRD4
nud	CHRNA4
nud	LYSMD3
nud	FMO1
nud	GABRA2
nud	FRMD4A
nud	SAGE1
nud	GRIK2
nud	WASF1
nud	KMO
nud	TCF7L2
nud	NTRK2
nud	RHOA
nud	CHRNG
nud	GABBR2
nud	RPTOR
nud	CRHR1
nud	OPRK1
nud	CHRNA10
nud	NF2
nud	ALDH2
nud	F2R
nud	ACHE
nud	GABARAP

nud	HTR3B
nud	HTR2A
nud	CYP2A6
nud	ERBB4
nud	ANAPC1
nud	CHRNB1
nud	NRG3
nud	CYP2B6
nud	GRM7
nud	PNPLA2
nud	FN1
nud	AHR
nud	CHRNB4
nud	PLG
nud	CHRNA3
nud	ZCCHC14
nud	CAD
nud	HPGDS
nud	PPARA
nud	ADH7
nud	DBH
nud	DNM1
nud	CSMD1
nud	OPRM1
nud	TH
nud	APP
nud	GRM5
nud	HTR3A
nud	LCP1
nud	CHRNA7
nud	ANKK1
nud	NR4A3
nud	NRXN3
nud	CHRNB2
nud	CYP2A13
nud	IL6
nud	PLA2G6
nud	CHRNA6
nud	CLPTM1L
nud	FTCD
nud	PON1
nud	COMT
nud	TTC12

nud	GRM2
nud	MGC57346-CRHR1
nud	DNMT3B
nud	TAAR1
nud	SHC3
nud	BDNF
nud	ADH1B
nud	SLCO3A1
nud	FMO3
nud	RAPGEF3
nud	GABBR1
nud	CHRNA5
nud	ADH1C
nud	DDC
nud	NFKB1
nud	TAS2R16
nud	CXCL8
nud	CHRM1
nud	CYP3A5
nud	GALR1
nud	GSTT1
nud	CD200R1
nud	TBPL1
nud	MAOA
nud	CHRFAM7A
nud	ACE
nud	KCNJ6
nud	PLEKHG1
nud	DRD5
nud	ACTB
nud	NAAA
nud	TPH2
nud	ARSD
nud	FAAH
oud	IL6
oud	DDX53
oud	MET
oud	C1QL1
oud	IL10
oud	HCRT
oud	AICDA
oud	ZGPAT
oud	DAPK3

oud	BCHE
oud	BDNF
oud	COMMD3
oud	GAL
oud	LGALS1
oud	CLMP
oud	MAT1A
oud	ACAT1
oud	CRH
oud	OPRM1
oud	GCG
oud	ACAD8
oud	DRD2
oud	TRPV1
oud	OPRL1
oud	PITX3
oud	TLR4
oud	CCKBR
oud	PIK3CD
oud	CLOCK
oud	NPSR1
oud	HCRTR1
oud	FADD
oud	PIK3CG
oud	TH
oud	AQP4
oud	CCL28
oud	MMP9
oud	GRIN2B
oud	GRIN2A
oud	NOS1
oud	NR3C1
oud	SLC1A1
oud	OPRM1
oud	PIK3CB
oud	ARL6IP5
oud	BDNF
oud	GRIA1
oud	MDH2
oud	HCRT
oud	TXN
oud	ZCRB1
oud	NPS

oud	PIK3CA
oud	CCK
oud	NR4A2
oud	BAX
oud	DNMT3A
oud	FGFR3
oud	CREB1
oud	BCL2
oud	SLC1A2
oud	CARTPT
oud	ANKS1B
oud	MC2R
oud	ELK3
oud	ZNF804A
oud	GRIN3B
oud	SIRT1
oud	GALR1
oud	SLC6A3
oud	NTSR1
oud	LRRFIP1
oud	LEP
oud	MAPK8
oud	DRD1
oud	EPHB1
oud	ADH1B
oud	PDYN
oud	GRK5
oud	HTR1B
oud	CSNK1E
oud	FOXO1
oud	DBH
oud	OPRM1
oud	FAAH
oud	ABCB1
oud	FLAD1
oud	DLG4
oud	NGF
oud	OR2AG1
oud	CNR1
oud	OPRK1
oud	ADIPOQ
oud	DRD4
oud	FAS

oud	AGBL4
oud	MUSK
oud	MAOA
oud	LOC400927-CSNK1E
oud	NR3C2
oud	NTS
oud	NTF3
oud	PENK
oud	DRD3
oud	RGS9
oud	CRHR1
oud	ANKK1
oud	RAPSN
oud	BRSK2
oud	ANTXR2
oud	AUTS2
oud	HNRNPH1
oud	SLC6A2
oud	SLC6A4
oud	CEBPG
oud	POMC
oud	DRD2
oud	F2R
oud	GABRA2
oud	CRHR2
oud	OPRD1
oud	HTR3B
oud	COMT
oud	HTR3A
oud	CRHBP
oud	GRIN2B
oud	ZNF611
oud	SERPINA3
oud	BDNF
oud	ABAT
oud	ENO2
oud	GDNF
oud	NCAM1
oud	NTF4
oud	ALDH2
oud	EEF1A2
oud	GLRA1
oud	TPH2

oud	GABRB3
oud	NPY1R
oud	SAGE1
oud	GRM3
oud	GRIN2A
oud	CDK5
oud	HTR2A
oud	PER3
oud	MPDZ
oud	GABRB2
oud	GAD1
oud	TPH1
oud	GRIN3A
oud	CLOCK
oud	FKBP5
oud	CTNNA2
oud	RETN
oud	GRM2
oud	HNRNPH2
oud	CCDC42
cvd	REN
cvd	NOS1
cvd	TCP1
cvd	COL18A1
cvd	EFEMP2
cvd	MMP9
cvd	GPR18
cvd	RPS19
cvd	CTSB
cvd	NOS2
cvd	CAMK2D
cvd	ACE
cvd	NOS3
cvd	TSPO
cvd	SLC6A4
cvd	POSTN
cvd	ADIPOQ
cvd	NR3C2
cvd	GPER1
cvd	LGALS3
cvd	TNFRSF11B
cvd	NT5E

Table S2: All KEGG pathways used in this study and its disorder

source	kegg
benchmarked brain	hsa04080
benchmarked brain	hsa04720
benchmarked brain	hsa04721
benchmarked brain	hsa04722
benchmarked brain	hsa04723
benchmarked brain	hsa04724
benchmarked brain	hsa04725
benchmarked brain	hsa04726
benchmarked brain	hsa04727
benchmarked brain	hsa04728
benchmarked brain	hsa04730
benchmarked brain	hsa05010
benchmarked brain	hsa05012
benchmarked brain	hsa05014
benchmarked brain	hsa05016
benchmarked brain	hsa05017
benchmarked brain	hsa05020
benchmarked brain	hsa05022
benchmarked brain	hsa05030
benchmarked brain	hsa05031
benchmarked brain	hsa05032
benchmarked brain	hsa05034
benchmarked cvd	hsa04010
benchmarked cvd	hsa04060
benchmarked cvd	hsa04062
benchmarked cvd	hsa04064
benchmarked cvd	hsa04151
benchmarked cvd	hsa04260
benchmarked cvd	hsa04350
benchmarked cvd	hsa04657
benchmarked cvd	hsa04668
benchmarked cvd	hsa04933
benchmarked cvd	hsa05323
benchmarked cvd	hsa05418
aud	hsa00620
aud	hsa04014
aud	hsa04015
aud	hsa04020
aud	hsa04024
aud	hsa04061

aud	hsa04080
aud	hsa04620
aud	hsa04625
aud	hsa04657
aud	hsa04726
aud	hsa04728
aud	hsa04742
aud	hsa04936
aud	hsa05030
aud	hsa05031
aud	hsa05032
aud	hsa05034
aud	hsa05133
aud	hsa05135
aud	hsa05140
aud	hsa05142
aud	hsa05143
aud	hsa05144
aud	hsa05146
aud	hsa05152
aud	hsa05164
aud	hsa05171
aud	hsa05321
aud	hsa05323
aud	hsa05417
aud	hsa05418
cud	hsa01521
cud	hsa01522
cud	hsa04012
cud	hsa04014
cud	hsa04015
cud	hsa04020
cud	hsa04022
cud	hsa04024
cud	hsa04062
cud	hsa04066
cud	hsa04068
cud	hsa04072
cud	hsa04080
cud	hsa04151
cud	hsa04261
cud	hsa04360
cud	hsa04370

cud	hsa04371
cud	hsa04380
cud	hsa04540
cud	hsa04550
cud	hsa04620
cud	hsa04662
cud	hsa04668
cud	hsa04713
cud	hsa04720
cud	hsa04722
cud	hsa04723
cud	hsa04724
cud	hsa04725
cud	hsa04726
cud	hsa04727
cud	hsa04728
cud	hsa04730
cud	hsa04750
cud	hsa04915
cud	hsa04917
cud	hsa04921
cud	hsa04926
cud	hsa04928
cud	hsa04930
cud	hsa04934
cud	hsa04935
cud	hsa04960
cud	hsa05022
cud	hsa05030
cud	hsa05031
cud	hsa05032
cud	hsa05034
cud	hsa05135
cud	hsa05161
cud	hsa05167
cud	hsa05205
cud	hsa05207
cud	hsa05210
cud	hsa05212
cud	hsa05213
cud	hsa05214
cud	hsa05215
cud	hsa05218

cud	hsa05220
cud	hsa05221
cud	hsa05224
cud	hsa05226
cud	hsa05235
nud	hsa00350
nud	hsa00380
nud	hsa00980
nud	hsa00982
nud	hsa04024
nud	hsa04072
nud	hsa04080
nud	hsa04725
nud	hsa04726
nud	hsa04728
nud	hsa04742
nud	hsa04936
nud	hsa05030
nud	hsa05031
nud	hsa05032
nud	hsa05034
nud	hsa05135
nud	hsa05207
oud	hsa00350
oud	hsa00380
oud	hsa01521
oud	hsa01522
oud	hsa01524
oud	hsa04014
oud	hsa04015
oud	hsa04020
oud	hsa04024
oud	hsa04068
oud	hsa04080
oud	hsa04151
oud	hsa04152
oud	hsa04210
oud	hsa04211
oud	hsa04668
oud	hsa04722
oud	hsa04724
oud	hsa04726
oud	hsa04728

oud	hsa04750
oud	hsa04915
oud	hsa04930
oud	hsa04932
oud	hsa04933
oud	hsa04936
oud	hsa05017
oud	hsa05020
oud	hsa05030
oud	hsa05031
oud	hsa05034
oud	hsa05142
oud	hsa05161
oud	hsa05162
oud	hsa05167
oud	hsa05215
cvd	hsa00220
cvd	hsa00330
cvd	hsa04924
cvd	hsa04926
cvd	hsa05415

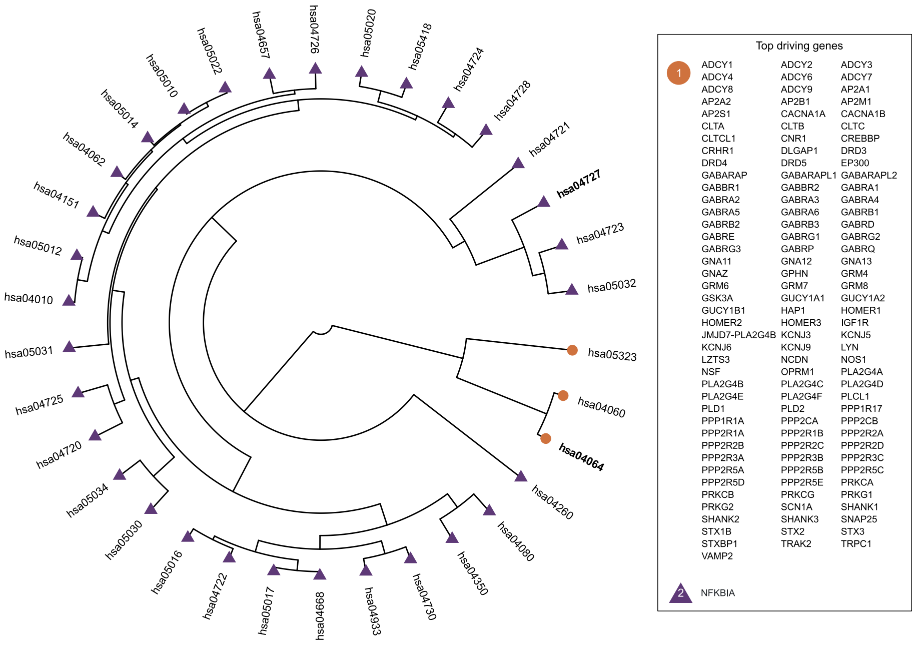

Figure S1: Heirarchical clustering for benchmarked data. Clusters are color and shaped coordinated. As shown, pathways hsa04064 (NF-kappa B signaling pathway) and hsa04727 (GABAergic synapse) are bolded as they have the highest Jaccard similarity.

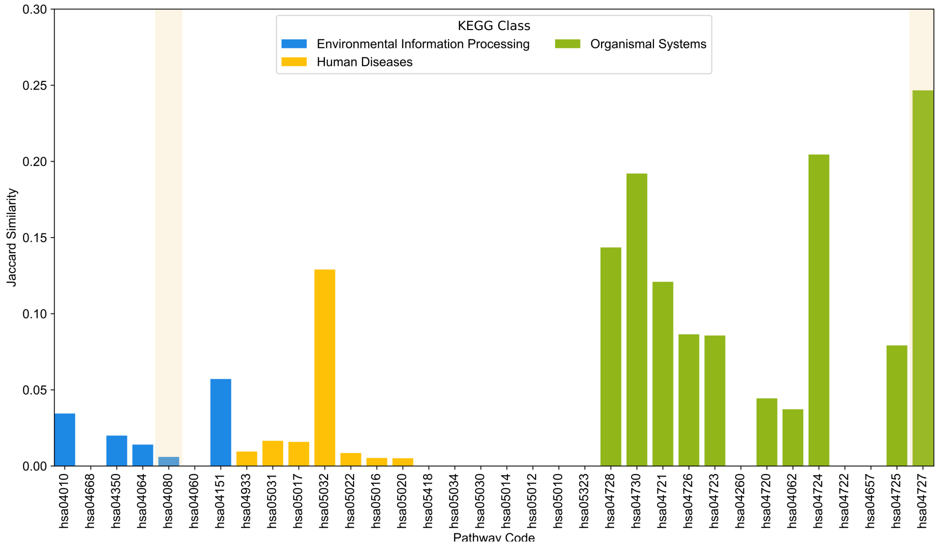

Figure S2: All Jaccard similarity results for benchmarked data. Pathways that cluster separately are highlighted in yellow. As shown, environmental information processing and organismal systems were the classes of two pathways that diverged from the rest.
